# Assessment of the aggregate index of systemic inflammation in dyslipidemia with dry eye disease: a retrospective case-control

**DOI:** 10.3389/fopht.2026.1762122

**Published:** 2026-06-11

**Authors:** Amani Y. Alhalwani, Asma Altasan, Nawaf Alasmari, Asma Almutiri, Mustafa Sunbul, Razan Khalifa, Jumanah Makhtoum, Shatha Jambi

**Affiliations:** 1 College of Science and Health Professions, King Saud bin Abdulaziz University for Health Sciences, Jeddah, Saudi Arabia; 2 Department of Biomedical Research, King Abdullah International Medical Research Center, Jeddah, Saudi Arabia; 3 College of Applied Medical Sciences, King Saud bin Abdulaziz University for Health Sciences, Jeddah, Saudi Arabia; 4 Ministry of the National Guard-Health Affairs, Jeddah, Saudi Arabia; 5 College of Medicine, Umm Al-Qura University, Mecca, Saudi Arabia; 6 College of Medicine, King Faisal University, Hofuf, Saudi Arabia; 7 Faculty of Medicine, King Abdul Aziz University, Jeddah, Saudi Arabia

**Keywords:** aggregate index of systemic inflammation ratio, C-reactive protein, dry eye disease, dyslipidemia, inflammation

## Abstract

**Background:**

Dyslipidemia (DLP) is characterized by high rates of occurrence of inflammation. One outcome of DLP development is dry eye disease (DED). The level of inflammation was assessed by identifying clinically relevant parameters in the complete blood count (CBC). In this study, the aim was to evaluate inflammation in DLP-DED patients using the aggregate index of systemic inflammation (AISI) and inflammatory biomarkers, including C-reactive protein (CRP).

**Methods:**

A total of 214 patients who had been clinically diagnosed with DLP at King Abdulaziz Medical City between 2016 and 2023 were randomly selected for retrospective analysis. The patients were allocated to one of two groups: DLP only as the control group and DLP with DED as the study group. Blood biomarker tests and demographic data were gathered and analyzed. The means, standard deviations, and medians of the blood results were calculated. Associations with lipid profile and inflammatory indicators were evaluated by Spearman’s correlation analysis. Moreover, a multivariable logistic regression analysis was performed to assess the independent association between AISI and DED, controlling for age, gender, and body mass index (BMI).

**Results:**

The DLP-DED group demonstrated higher neutrophil and monocyte counts relative to the DLP group. In the DLP-DED group, AISI showed a positive correlation with CRP and a weak negative correlation with HDL and total cholesterol.

**Conclusions:**

AISI may indicate components of systemic inflammation in patients with dyslipidemia; however, it was not associated with dry eye disease following adjustment for confounding variables. Additional evaluation of its clinical value requires prospective research.

**Impact statement:**

Investigating inflammatory biomarkers in patients with dyslipidemia and dry eye disease may improve understanding of the pathogenesis and pave the way for novel therapeutic approaches to alleviate this debilitating condition.

## 1 Introduction

Dyslipidemia (DLP) occurs when there are irregularities in the levels of blood lipids, such as elevated levels of total or low-density lipoprotein (LDL) cholesterol, triglycerides, or low levels of high-density lipoprotein (HDL) cholesterol. These irregularities may occur either singly or in combination (1). DLP is also characterised by high levels of inflammation. This condition is becoming increasingly apparent in Saudi Arabia; the World Health Survey -Saudi Arabia showed that in 2022 DLP was prevalent in 43% of the Saudi population (2). According to a study published in the *American Journal of Ophthalmology*, people with DLP have a higher risk of developing dry eye disease (DED) than those without DLP. Increased levels of triglycerides, LDL cholesterol and total cholesterol significantly increase the risk and progression rate of DED. This study sheds light on the relationship between DLP and DED and emphasises the need to control lipid levels to prevent and treat DED (3). DED is a common, painful inflammatory disorder of tear film that occurs among adults worldwide. It is characterised by discomfort, blurred vision and tear-film instability, which lead to ocular surface injury, inflammation, and increased tear-film osmolarity (4).

DED prevalence is on the rise. In Saudi Arabia, a study reported that the ages of 541 people who had the disease ranged from one to 72 years with a median age of 32 years; a majority (85.3%) of the group lived in the country’s western region and 70.9% of the respondents were women (5). DED is classified into two subtypes: one involves decreased tear secretion (aqueous-deficient DED), and the other, increased tear evaporation (hyper-evaporation DED). Both subgroups are linked to systemic risk factors. Various factors have been found to contribute significantly to the development of DED: menopause, anxiety, systemic rheumatological diseases, use of anxiolytics, daily medication, ocular surgery, poor diet quality, increased consumption of ultra-processed food, lack of caffeine intake, prolonged exposure to air conditioning, and experience of certain chronic diseases such as DLP, diabetes, and other inflammatory disorders (6).

Researchers have examined the correlation between inflammation, DED and DLP. A recent study examined how DED-related damage to the ocular surface could trigger an inflammation response that initially appeared as an acute and self-limiting reaction. However, inflammation may become chronic if the underlying problem is not resolved. DED may occur in cases in which inflammation affects the tear glands and typical ocular structures. After DED develops, inflammation and DED interact and reinforce one another. Therefore, inflammation can be seen as a DED cause and effect (7). The aggregate index of systemic inflammation (AISI), which comprises data for neutrophil, platelet, monocyte and lymphocyte levels is considered a biomarker by which systemic inflammatory conditions can be quantified through the study of whole blood cells (8). Research indicates that the AISI can be used as a biomarker for DLP (9).

Another study found a strong correlation between DED and DLP, especially in females, and total cholesterol and other lipid parameters were associated with DED (10). More investigation is required, particularly among the Saudi population, to understand thoroughly the relationship between underlying inflammation in DED and DLP (11). Moreover, cytokine and chemokine levels, which are used as inflammation biomarkers, were significantly higher in the tears of DED patients than in healthy controls. The mRNA levels for the four indicators that were measured in the study all increased, and their fold changes matched those of the cytokine concentrations discovered in the tear samples. In another study, DED in Korean women was associated with high serum cholesterol levels; this study highlights the significance of eye examinations and independent lipid profile monitoring in patients with DLP because of its possible correlation with DED progression.

This study aimed to assess the correlation between the aggregate index of systemic inflammation (AISI) and inflammatory level in DLP with DED; to investigate the biomarkers that were associated with DED; and to assess the correlation between AISI, the lipid profiles and the levels of C-reactive protein (CRP), neutrophils, monocytes, platelets and lymphocytes in all groups.

## 2 Methodology

### 2.1 Study population

This study was of a retrospective, case-control design with sequential sampling. It involved an analysis of data collected between 2016 and 2023. Patients with DLP were selected from the outpatient clinic of King Abdulaziz Medical City (KAMC) in Jeddah, Saudi Arabia. The patients were diagnosed with DED based on the primary physician’s assessment. However, there is insufficient available data on Schirmer’s test or tear break-up time in the hospital information system (Bestcare). The patients were divided into two groups: a control group, which comprised those with DLP alone; and a study group, which comprised those with DLP and DED.

### 2.2 Demographic information

The patients’ demographic information was collected from the Bestcare. This study involved the performance of tests for inflammatory biomarkers that included neutrophils, monocytes, platelet, lymphocytes, CRP, AISI, and lipid profile. The AISI was calculated according to the following equation: (neutrophils*monocytes* platelet)/lymphocytes) (5, 12). Patients who were under 18 years old, smokers, wearing contact lenses, had undergone eye surgery or had cancer were excluded from the study.

### 2.3 Sample size calculation

Sample size was calculated using Raosoft software by the website (https://raosoftcalculator.com/). The total number of dyslipidemia patients from January 2016 to June 2023 is around 15000. The required sample size will be estimated at a 95% confidence level, with a 40% prevalence of Dyslipidemia (13) and a margin of error of ±5%. The required minimum sample size will be determined to be 360. The sampling technique employed was convenient and yielded a representative sample of the population suffering from Dyslipidemia and dry eye disease. Due to the limited sample size, all patients involved in this study during the specified period were included, n=214. The sample size was influenced by the number of available patients who visited the clinic (151 with DLP alone and 63 with DLP-DED). Patients who were under 18 years old, smokers, contact-lens wearers, had undergone eye surgery, or had cancer were excluded from the study.

### 2.4 Data collection and analysis

The patient data were obtained from the hospital information system according to the codes of the World Health Organization’s International Classification of Diseases, 10^th^ edition, which were listed in the patients’ medical records. Patient’s demographics [age, Body mass index (BMI) and gender], medical history based on the available data in the electronic medical records, laboratory data including complete blood count parameters (neutrophils, lymphocytes, monocytes, platelets), C-reactive protein (CRP) and Lipid profile [triglycerides, high-density lipoprotein (HDL), low-density lipoprotein (LDL), and total cholesterol] were collected.

### 2.5 Statistical analysis

Statistical analyses were conducted utilizing GraphPad Prism (GraphPad Software Inc., San Diego, CA, USA). Continuous variables were evaluated for normal distribution. Due to the non-normal distribution of most variables, they are reported as median (interquartile range, IQR), with group comparisons conducted using the Mann-Whitney U test. Categorical variables were represented as frequencies and percentages and analyzed using the chi-square test.

Spearman’s rank correlation analysis was employed to assess the associations between AISI and lipid profile parameters, as well as between AISI and inflammatory markers, in both DLP and DLP-DED groups.

A multivariable logistic regression analysis was conducted to evaluate the independent relationship between AISI and dry eye disease (DED), controlling for age, gender, and body mass index (BMI). Adjusted odds ratios (aORs) accompanied with 95% confidence intervals (CIs) were computed. The Hosmer-Lemeshow goodness-of-fit test was used to evaluate calibration, and the area under the receiver operating characteristic curve (AUC) was used to evaluate model discrimination. Statistical significance was defined as a two-tailed *P*-value of less than 0.05.

## 3 Results

The initial study population was 220 patients; after exclusion, 214 patients were recruited and divided into two groups: the DLP-DED group (n= 63), and the DLP group (n= 151). The patients’ demographic data are listed in **Table 1**. First, we examined age; the median (IQR) age was significantly higher in the DLP-DED group relative to the DLP group [64.0 (55–70) compared. 50.5 (43–65) years, *P* < 0.0001]. then gender was considered; it was found to be almost equal in the DLP-DED group, 50.7% male and 49.3% female, while the DLP group was not evenly distributed: 43.7% male, and 56.3% female. This difference was not statistically significant (*P* = 0.345). Both groups were overweight, but the median BMI did not showing a significant difference between groups (27.10 [25.5-33.7] compared. 28.83 [25.8-32.8] kg/m², *P* = 0.351).

**Table 1:** Demographic data for the DLP-DED and DLP groups.

Parameters	DLP-DED (n=63)	DLP (n=151)	*P-value
Age (Years)			<0.0001
Mean (SD)	62.5 (10.22)	53.7 (15.01)	
Median	64.0	50.5	
IQR	55-70	43-65	
Gender, n (%)			0.345**
Male	32 (50.7)	66 (43.7)	
Female	31 (49.3)	85 (56.3)	
BMI Obesity ≥ 27 kg/m^2^			0.351
Mean (SD)	30.5 (7.4)	29.7 (10.1)	
Median	27.10	28.83	
IQR	25.5-33.7	25.8-32.8	

*Mann-Whitney test. BMI, body mass index.

**chi-square test.

One aim of this study was to aid in the early detection of patients’ inflammation status through the application of cost-effective tests such as the measurement of CBC and CRP levels in blood. The inflammation marker findings are summarised in **Table 2**. The median CRP level was 2.75 (1.25-16.4) mg/L in the DLP DED group and 5.25 (1.60-13.3) mg/L in the DLP group (*P* = 0.255).

**Table 2:** Inflammation marker findings for the DLP-DED and DLP groups.

Inflammation markers	DLP-DED (n=63)	DLP (n=151)	*P-value
CRP Normal range: 0–5 mg/L			0.255
Mean (SD)	9.1 (12.10)	14.6 (32.59)	
Median	2.75	5.25	
IQR	1.25-16.4	1.60-13.3	
Neutrophils Normal range: 2-7.50 x 10^9^/L			0.0340
Mean (SD)	3.8 (1.4)	3.7 (3.9)	
Median	3.91	3.06	
IQR	2.63-4.94	2.10-4.170	
Monocytes Normal range: 0.2-0.8 x 10^9^/L			0.0475
Mean (SD)	0.59 (0.20)	0.51 (0.16)	
Median	0.5400	0.4900	
IQR	0.45-0.73	0.39-0.60	
Platelets Normal range: 150–450 x 10^9^/L			0.800
Mean (SD)	269.2 (92.0)	267.7 (81.3)	
Median	250.0	265.0	
IQR	211.0-311	214.5-322	
Lymphocytes Normal range: 1.5-4.0 x 10^9^/L			0.191
Mean (SD)	2.80 (1.011)	2.53 (0.8615)	
Median	2.570	2.370	
IQR	2.11-3.39	1.98-3.06	
AISI Normal range:			0.222
Mean (SD)	238.5 (179.3)	220.6 (209.9)	
Median	191.8	151.2	
IQR	118.3-315.1	93.5-278.1	

*Mann-Whitney test. CRP, C-reactive protein; ESR, erythrocyte sedimentation rate; AISI, aggregate index of systemic inflammation.

The median neutrophil counts were markedly elevated in the DLP DED group [3.91 (2.63-4.94) as compared to 3.06 (2.10-4.17) ×10^9^/L, *P* = 0.034].

The median monocyte counts were elevated in the DLP DED group [0.54 (0.45-0.73) compared with 0.49 (0.39-0.60) ×10^9^/L, *P* = 0.047]. The calculated AISI values were <300; greater in the DLP-DED group. However, no significant differences were observed in platelet counts [250.0 (211.0-311) compared with 265.0 (214.5-322) ×10^9^/L, *P* = 0.800], lymphocyte counts [2.57 (2.11-3.39) compared with 2.37 (1.98-3.06) ×10^9^/L, *P* = 0.191], or AISI values [191.8 (118.3-315.1) compared with 151.2 (93.5-278.1), *P* = 0.222] yet, this difference was not statistically significant.

**Figure 1:**
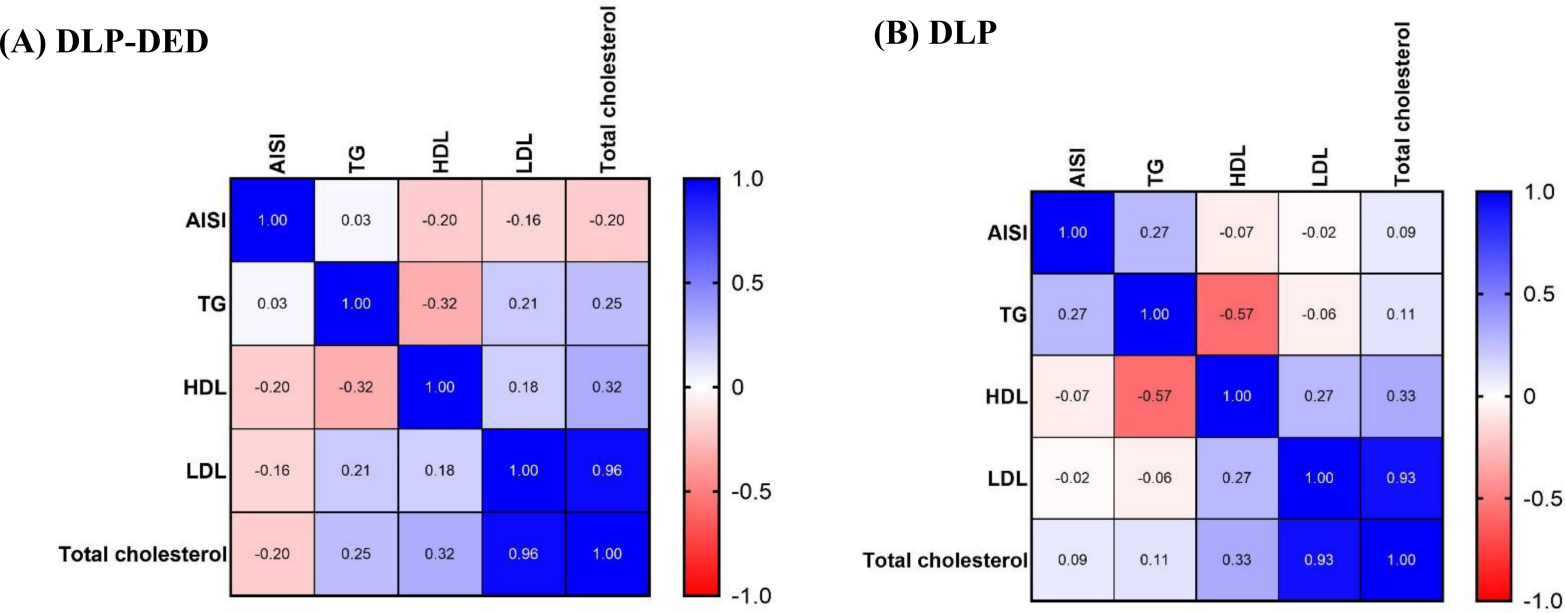
Spearman’s rank correlation heatmaps illustrating the correlation between AISI and lipid profile indicators in dyslipidemic-related patients. **(A)** DLP-DED group and **(B)** DLP group. Heatmaps illustrate Spearman’s correlation coefficients (r) between the aggregate index of systemic inflammation (AISI) and variables of the lipid profile, particularly triglycerides (TG), high-density lipoprotein (HDL), low-density lipoprotein (LDL), and total cholesterol. Color intensity indicates the magnitude and orientation of correlation (blue, positive correlation; red, negative correlation).

The laboratory findings for lipid profile variables in the DLP-DED and DLP patients are summarised in **Table 3**. No statistically significant differences were detected between the DLP-DED and DLP groups regarding triglycerides [1.300 (0.89-1.88) compared with 1.210 (0.86-1.66) mmol/L, *P* = 0.425], HDL [1.11 (1.03-1.51) compared with 1.19 (0.99-1.42) mmol/L, *P* = 0.983], LDL [2.92 (2.43-3.84) compared with 3.05 (2.21-4.02) mmol/L, *P* = 0.728], or total cholesterol [4.88 (4.12-5.71) compared to 4.88 (3.94-5.93) mmol/L, *P* = 0.768]. The Spearman rank correlation analysis between AISI and lipid profile parameters is summarised in **Table 4** and illustrated in **Figure 1**. In the DLP−DED group, AISI showed a weak negative correlation with HDL (*r* = -0.20, *P* = 0.029) and total cholesterol (*r* = -0.20, *P* = 0.028), although the correlation with LDL was not statistically significant (*r* = -0.16, *P* = 0.08). There are no significant correlated relationships between AISI and lipid markers in the DLP group. Triglycerides showed a negative association with HDL in both groups, with a stronger correlation in the DLP group (r = -0.57, < 0.001) than in the DLP-DED group (*r* = -0.32, < 0.001). LDL revealed a significant positive correlation correlated with total cholesterol in both groups (*r* = 0.96 and *r* = 0.94, respectively; < 0.001).

**Table 3:** Lipid profile findings for the DLP-DED and DLP groups.

Lipid profile	DLP-DED (n=63)	DLP (n=151)	*P-value
Triglyceride Normal range: <1.70 mmol/L			0.425
Mean (SD)	1.463 (0.75)	1.402 (0.98)	
Median	1.300	1.210	
IQR	0.89-1.88	0.86-1.66	
HDL Normal range: 1.55 ~10 mmol/L			0.983
Mean (SD)	1.233 (0.2998)	1.219 (0.3161)	
Median	1.11	1.19	
IQR	1.03-1.51	0.99-1.42	
LDL Normal range: <2.59 mmol/L			0.728
Mean (SD)	3.00 (0.99)	3.28 (1.765)	
Median	2.92	3.05	
IQR	2.43-3.84	2.21-4.018	
Total cholesterol Normal range: ~5.18 mmol/L			0.768
Mean (SD)	4.895 (1.08)	5.125 (1.85)	
Median	4.88	4.88	
IQR	4.12-5.71	3.94-5.93	

*Mann-Whitney test. Abbreviations: HDL, high-density lipoprotein; LDL, low-density lipoprotein.

**Table 4:** Spearman’s rank correlation analysis in DLP-DED and DLP groups.

Variables	DLP-DED (*r*)	DLP-DED (*P*)	DLP (*r*)	DLP (*P*)
AISI vs TG	0.03	0.76	0.27	0.10
AISI vs HDL	-0.20	0.029*	-0.07	0.68
AISI vs LDL	-0.16	0.08	-0.02	0.90
AISI vs Total cholesterol	-0.20	0.028*	0.09	0.62
TG vs HDL	-0.32	<0.001***	-0.57	<0.001***
TG vs LDL	0.21	0.024*	-0.06	0.72
TG vs Total cholesterol	0.25	0.005**	0.11	0.52
HDL vs LDL	0.18	0.047*	0.27	0.11
HDL vs Total cholesterol	0.32	<0.001***	0.33	0.042*
LDL vs Total cholesterol	0.96	<0.001***	0.94	<0.001***

*< 0.05, **< 0.01, ***< 0.001. Values represent Spearman’s correlation coefficients (r) together with their corresponding P-values. DLP-DED, dyslipidemia with dry eye disease; DLP, dyslipidemia without dry eye disease; AISI, aggregate index of systemic inflammation; TG, triglycerides; HDL, high-density lipoprotein; LDL, low-density lipoprotein.

**Figure 2:**
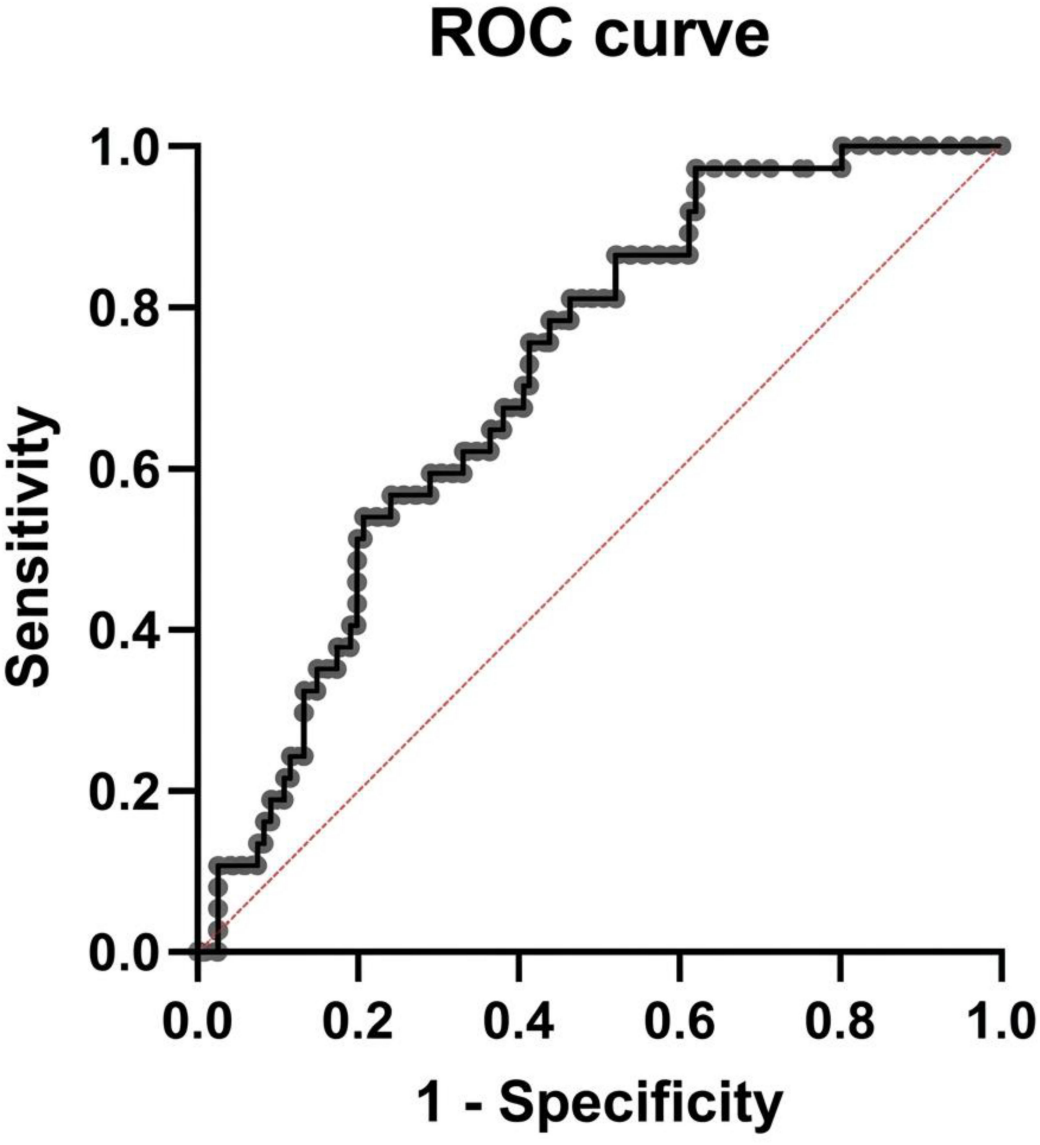
Receiver operating characteristic (ROC) curve of the multivariable logistic regression model for dry eye disease (DED). The model included age, gender and body mass index (BMI), and the aggregate index of systemic inflammation (AISI). The area under the curve (AUC) was 0.716 (95% CI 0.631–0.801), indicating acceptable discriminatory performance.

In the multivariable logistic regression model, after adjusting for variables for age, gender, BMI, and AISI, only age was independently associated with DED **Table 5**. Every one-year increase in age is associated with a 5% rise in the probability of DED [(aOR) 1.05, 95% (CI) 1.02–1.08, < 0.001]. Following adjustment, gender, BMI, and AISI showed no significant association with DED. The model demonstrated acceptable discriminatory performance (AUC = 0.716, 95% CI: 0.631–0.801) and adequate calibration, as indicated by the Hosmer–Lemeshow goodness-of-fit test (*P* = 0.21) (**Figure 2**).

**Table 5:** Multivariable logistic regression analysis of factors associated with dry eye disease (Adjusted).

Variable	Adjusted odds ratio (aOR)	95% confidence interval	*P-value
Age	1.050	1.021 - 1.083	<0.001*
Female (vs male)	0.72	0.33 -1.58	0.41
BMI (kg/m²)	0.96	0.90 - 1.03	0.27
AISI (per unit increase)	1.000	0.998 - 1.002	0.67

*Statistically significant at < 0.05. Multivariable logistic regression model including age, gender, BMI, and AISI. Odds ratios are adjusted for all variables listed in the table. CI, confidence interval; BMI, body mass index; AISI, aggregate index of systemic inflammation.

## 4 Discussion

In this study, a significant difference (P<0.001) was found in the mean ages of the DLP and DLP-DED groups. This is in agreement with previous studies, which show that DLP and DED incidence is substantial in the elderly population (14, 15). Other studies show similarly that midDLPe to elderly age is a risk factor for DED (9, 16).

In this study, females were predominant in the DLP group, which was comparable with previous research (8, 17, 18). Surprisingly, the numbers of males and females in the study group DLP-DED were roughly equal, whereas previous studies have shown DED incidence to be higher among females than among males. This was shown in studies performed in Saudi Arabia (19), Dubai (20), and among young people in Poland (21).

In addition, it has been reported previously that higher BMI is linked with DLP patients (22). We observed no significant difference between the BMIs in the DLP and DLP-DED groups, as the medians were within the overweight classification for both groups. Excess body weight could be an independent risk factor for both DLP and DED, as excess body weight exacerbates body inflammation (23, 24).

In this study, the levels of inflammatory blood biomarkers were more severe in DLP patients than in DLP-DED patients. Still, the CRP level in both groups exceeded the normal range. This finding is similar to the reported finding of inflammation (25).

Neutrophil and monocyte counts were found to be significantly higher in the DLP-DED group when compared with the DLP patients. There were no differences in platelet or lymphocyte numbers between the groups. Our study findings were supported by a previous study, which showed that high numbers of neutrophils and monocytes were associated with disease severity in eye tears among DED patients, as inflammation is involved in killing and digesting bacteria (26). Another study supported our findings, as researchers found that the neutrophil-to-lymphocyte ratio (NLR) was significantly higher among DED patients than healthy controls, whereas the platelet-to-lymphocyte ratio (PLR) did not differ significantly. The researchers concluded that the NLR could be used as a predictor of inflammation to estimate the severity of DED (27).

The AISI values were insignificantly greater in the DLP-DED than in the DLP patients. This finding is consistent with the fact that AISI is used to indicate the development of age-related macular degeneration, which shows that AISI is linked to inflammation status (11). A recent ocular study demonstrated that AISI was associated with inflammatory diseases, and the researchers in this case pointed out that inflammation was essential to protect the immune system by scavenging pathogens and diseases (28).

The systemic inflammation CBC indicators in elderly and non-elderly patients were insignificantly associated with DED incidence, as has been shown in previous studies (29, 30). The correlation analysis in this study showed that risk factors for DLP were increased age, overweight, and inflammation as indicated by increased levels of inflammation markers.

A previous study showed the impact of body lipid components on the tear film. Lipids are secreted mainly from the meibomian gland to maintain the ocular surface and to prevent tears from evaporating from the ocular surface (31). A study reported that irregularity of the tear film caused increased stress on the ocular surface, which in turn led to DED, and if left untreated, this might result in perforation of the cornea, visual impairment, and blindness (32). In a previous study, it was stated that the lipid profile components contributed to abnormalities in levels of total blood cholesterol, HDL, LDL, and triglyceride. Interestingly, in this study, all lipid profile components were within normal ranges except LDL.

In another study, patients with DLP were found to have an altered meibomian lipid composition with a notable increase in the quantity of cholesterol, which caused DED.

The change in lipid composition could reflect the physiological status of the tear film and could be used to interpret the pathogenesis of DED in DLP. Guliani et al. (36) reported that in patients with dysfunctional meibomian glands, HDL was the primary factor driving elevated total blood cholesterol levels. High levels of LDL and total cholesterol, and low levels of HDL, have been shown to be independent risk factors for vascular pathological events among DLP patients (33). Rathnakumar et al. (9) reported changes in lipid profile levels that coincided with the development of DLP and DED. Elevated levels of CRP and total cholesterol as inflammation biomarkers have also been associated with DED patients (34).

In this study, there was a positive correlation between the AISI and levels of inflammation markers in the DLP-DED group. This was similar to the findings of another study, which showed that the AISI was positively correlated with other inflammation biomarkers (35). Unexpected DLP: there was an insignificant negative correlation between AISI and ESR in the study group. This insignificant correlation could be related to our small sample size or the nature of our enrolled patients, whose DLP seemed to be highly controlled by medications and extensive healthcare.

The limitation of this study is its retrospective design, which inherently limited the reliability and reproducibility of its findings regarding DED. Additionally, specific patient DED types or other disease indicators beyond the doctor’s diagnosis—such as essential ocular examinations like the Schirmer’s test and tear break-up time—were not included because they were unavailable in the hospital system. The absence of data from healthy individuals further compromised the study’s validity. Additionally, we were unable to adequately account for potential confounding variables, such as dietary habits, medication use (e.g., statins or anti-inflammatory drugs), lifestyle factors, or comorbidities, including diabetes.

Future work is advised, including prospective longitudinal or interventional studies that incorporate a broader array of inflammatory markers and diverse populations, to validate these findings and explore potential mechanisms in greater depth. Furthermore, a future study is recommended to investigate the association between medication use, comorbidities, and lifestyle factors, which will aid in developing treatment plans and management strategies.

## 5 Conclusions

This investigation noticed differences in specific inflammatory markers between patients with DLP and those with DLP-DED. Patients with DLP-DED demonstrated elevated neutrophil and monocyte counts, indicating a more significant systemic inflammatory profile. AISI showed weak correlations with specific inflammatory and lipid indicators; however, it was not independently associated with DED after adjustment for age and other variables. These data suggest a possible role of systemic inflammation in the co-occurrence of dyslipidemia and dry eye syndrome. Additional prospective studies are necessary to further clarify the clinical significance of AISI and to investigate the impact of lipid and inflammatory changes in the pathogenesis of DED in individuals with DLP. Examining the lipid composition of the tear film may yield further mechanistic understanding.

## Data Availability

The raw data supporting the conclusions of this article will be made available by the authors, without undue reservation.
